# Timely-Automatic Procedure for Estimating the Endocardial Limits of the Left Ventricle Assessed Echocardiographically in Clinical Practice

**DOI:** 10.3390/diagnostics10010040

**Published:** 2020-01-13

**Authors:** Darian M. Onchis, Codruta Istin, Cristina Tudoran, Mariana Tudoran, Pedro Real

**Affiliations:** 1 Department of Computer Science, West University of Timisoara, 300223 Timisoara, Romania; 2 Department of Computers and Information Technology, Politehnica University of Timisoara, 300006 Timisoara, Romania; istin.codruta@gmail.com; 3 Department VII, Internal Medicine II, Discipline of Cardiology, Victor Babes University of Medicine and Pharmacy, 300041 Timisoara, Romania; cristina13.tudoran@gmail.com (C.T.); mariana.tudoran@gmail.com (M.T.); 4 Department of Applied Mathematics I, University of Seville, 41004 Sevilla, Spain; real@us.es

**Keywords:** echocardiographic diagnostics, left ventricle wall, Gabor–Hough algorithm

## Abstract

In this paper, we propose an analytical rapid method to estimate the endocardial borders of the left ventricular walls on echocardiographic images for prospective clinical integration. The procedure was created as a diagnostic support tool for the clinician and it is based on the use of the anisotropic generalized Hough transform. Its application is guided by a Gabor-like filtering for the approximate delimitation of the region of interest without the need for computing further anatomical characteristics. The algorithm is applying directly a deformable template on the predetermined filtered region and therefore it is responsive and straightforward implementable. For accuracy considerations, we have employed a support vector machine classifier to determine the confidence level of the automated marking. The clinical tests were performed at the Cardiology Clinic of the County Emergency Hospital Timisoara and they improved the physicians perception in more than 50% of the cases. The report is concluded with medical discussions.

## 1. Introduction

In clinical cardiology, echocardiography has become nowadays one of the most employed diagnostic procedures [1,2]. Sustained progresses in the field of acoustic engineering and in digital image processing technology have conferred to echocardiography increased availability and diagnostic accuracy, while this method remains the most inexpensive and convenient diagnostic modality, also being the most time-saving one. Thus, some of the inherent impediments related to cardiac ultrasonography remains partially unsolved [2]. There are several clinical situations where it is technically difficult to acquire an appropriate image, such as in patients with poor ultrasonographic window, e.g. those with obesity, pulmonary diseases, surgical scars and drains, mechanical ventilation, etc. [3]. In these cases, errors associated with the study procedure such as poor image quality, artefacts, and measurement errors render the endocardial border delineation (EBD), which is an important phase for the evaluation of volumes, unreliable. This leads mostly to underestimation of volumes and errors in the assessment of some valuable hemodynamic parameters, especially of the left ventricular (LV) ejection fraction (EF), that is essential for heart failure’s classification, for establishing an optimal therapeutic approach, and for prognostic purpose. Several attempts to overcome these challenges have been made. At first, the international societies of echocardiography have developed rigorous protocols for the acquirement and measurement of cardiac structures [2], and then, transesophageal ultrasonography (TEE) or the use of contrast agents for an improved EBD were recommended [3]. Other possibilities, in suboptimal echocardiographic assessments, are cardiac magnetic resonance imaging (MRI), single-photon emission computed tomography (SPECT), or invasive methods, causing increased medical cost and healthcare system burden [4]. Finally, progresses in the field of digital image processing attempted to provide alternative solutions [5,6]. There are many studies in digital image processing on how to enhance the marking of the left ventricular wall. Among the most accurate ones are the procedures based on determining volumes from three-dimensional echocardiography (e.g., [5]) or on an active appearance model of the left ventricle with manual correction (e.g., [6]). In this paper, we introduce a complete timely-automatic procedure for the delimitation of the left ventricular wall. The procedure consists in employing an anisotropic Hough transform over a Gabor-like filtered region of interest through the use of guided deformable template for automatically marking a precise approximation of the left ventricular wall. The algorithm is rapid and accurate and therefore suitable for clinical integration via a pipeline of sequential processing made of the actual ultrasound investigation followed immediately by the computer processing.

The paper is structured as follows. In the next section, we present a mathematical brief where we review the necessary concepts on the Hough transform and the Gabor-like filtering. Section 3 introduces the main recognition algorithm together with the medical considerations regarding its applications. In the last section, we test the complete procedure and draw the medical conclusions in the form of a discussion.

## 2. Mathematical Brief

Hough transform (HT) is one of the most suitable techniques used in image analysis to extract features from an image [7,8,9,10,11]. The method was patented by [12] in 1962 for the detection of lines in photographs. The functioning of the algorithm lies in a proper choice of the parameters space for the set of lines on the plane.

Consider the implicit equation of a line, as expressed in [13]

(1) $$\begin{array}{c} f\left(\left(\hat{m},\hat{c}\right),\left(x,y\right)\right)=y-\hat{m}x-\hat{x}=0 \end{array}$$

where *m* and *c* are the slope and the intercept that characterizes the line. Equation (1) maps every pair of parameters $\left(\hat{m},\hat{c}\right)$ to a specific line, a shape. By swapping the roles of the parameters and the points and fixing one point, the equation defines all the lines that pass to the fixed point. Thus, if we consider some points in an image, they will be collinear if we can find a parameter $\left(\hat{m},\hat{c}\right)$ which satisfies Equation (1) for all the these fixed points. Following the work of Ballard [14], consider now a generic curve in analytic form

(2) $$\begin{array}{c} f\left(x,a\right)=0 \end{array}$$

where x is an image point and a is a parameter vector. For every point, we will find a manifold of parameters that satisfy Equation (2). The curve will be detected in the intersection of these manifolds. For example, if we consider a circumference

$$\begin{array}{c} \left(x-x_{0}\right)+\left(y-y_{0}\right)=r_{0}^{2}, \end{array}$$

because we can extract one parameter from the others, the number of free parameters is two. Every point $\left(x,y\right)$ will produce a cone in the parameter space $\left(a,b,r\right)$; if we consider three points on the plane, we can find the related circumference as in the intersection of these three cones. Now, we can add further information that can be extracted in an image: the gradient and the tangent on the points of the edge. We can use the equation of the curve together with its derivative to find all the parameters which satisfy $\frac{\partial f}{\partial x}\left(x,a\right)=0$ or similarly $\frac{\partial y}{\partial x}=\tan\left(\phi \left(x\right)-\frac{\pi }{2}\right)$, which is known since ϕ(x) is the gradient direction. Going back to the example of the circle, fixing the gradient with the point means that the center must lie *r* unit along the direction of the gradient, thus the number of free parameters reduces to one.

To develop a method for the recognition of a generic template in an image, Ballard [14] used the following parameters for a shape

(3) $$\begin{array}{c} a=\left\{y,S,\theta \right\} \end{array}$$

where $y=\left(x_{r},y_{r}\right)$ is a reference point, $S=\left(S_{x},S_{y}\right)$ are scale values for the orthogonal direction, and θ represents the orientation. The reference point y is described in terms of a table, called R-table, of possible edge pixel orientation. The other parameters are described in terms of transformation of the aforementioned table. The key for generalizing the Hough GHT transform is to use the directional information. Given a template, i.e., a set of boundary points $\left\{x_{B}\right\}$, a reference point *y* is chosen. After the discretization of the straight angle in subinterval 0,Δt,2Δt,…, for every boundary point, the gradient direction $\phi (x_{B})$ is computed, and then $r=y-x_{B}$ is stored in the bin of the R-Table corresponding to nΔt such that $\;\mathrm{mod}\;(\phi \left(x_{B}\right),\pi )\in n\Delta t$. Usually, to control the error in the computation of *r*, the barycenter of the boundary point is chosen as reference point and *r* is stored in polar coordinates.

To detect the region of interest of the best match in ultrasound image, we apply a Gabor filter to obtain local characteristics of the waves intensities [21,22,23,24]. Gabor filters are a good model of simple cells in visual cortex. The real part of a Gabor filter assumed to be centered at zero is the product of a sinusoid and a Gaussian:

(4) $$\begin{array}{c} g\left(x,y;\lambda ,\theta ,\phi ,\gamma \right)=\exp\left(-\frac{x^{\prime 2}+\gamma^{2}y^{\prime 2}}{2\sigma^{2}}\right)\cos\left(2\pi \frac{x^{\prime }}{\lambda }+\phi \right), \end{array}$$

where

(5) $$\begin{array}{cc} x^{\prime } & =x\cos\theta +y\sin\theta \end{array}$$

(6) $$\begin{array}{cc} y^{\prime } & =-x\sin\theta +y\cos\theta . \end{array}$$

We explain below the parameters of the filter. The wavelength given by the number of cycles/pixel is λ. The angle of the normal to the sinusoid is θ and it gives us the orientation. The phase given by the offset of the sinusoid is ϕ. Ellipticity is produced with gamma<1.

The spatial envelope of the Gaussian, σ, is controlled by the bandwidth, which at unity gives σ=0.56λ.

The half-response spatial frequency bandwidth *b* in octaves of a Gabor filter is related to the ratio σ/λ:

(7) $$\begin{array}{cc} b & =\log_{2}\frac{\left(\sigma /\lambda \right)\pi +\sqrt{\log2/2}}{\left(\sigma /\lambda \right)\pi -\sqrt{\log2/2}} \end{array}$$

(8) $$\begin{array}{cc} \frac{\sigma }{\lambda } & =\left(1/\pi \right)\sqrt{\log2/2}\frac{2^{b}+1}{2^{b}-1}. \end{array}$$

The value of σ cannot be specified directly. It can only be changed through the bandwidth *b*. The bandwidth value must be specified as a real positive number. The default is 1, in which case σ and λ are connected as follows: σ=0.56λ. The smaller is the bandwidth, the larger are σ, the support of the Gabor function, and the number of visible parallel excitatory and inhibitory stripe zones.

## 3. Materials and Methods

We describe in this section the complete recognition procedure for estimating the delimitation of the left ventricular wall as we implemented and tested it. To obtain the algorithm, we performed a series of sequential steps that led us to its refined formulation.

**Figure 1:**
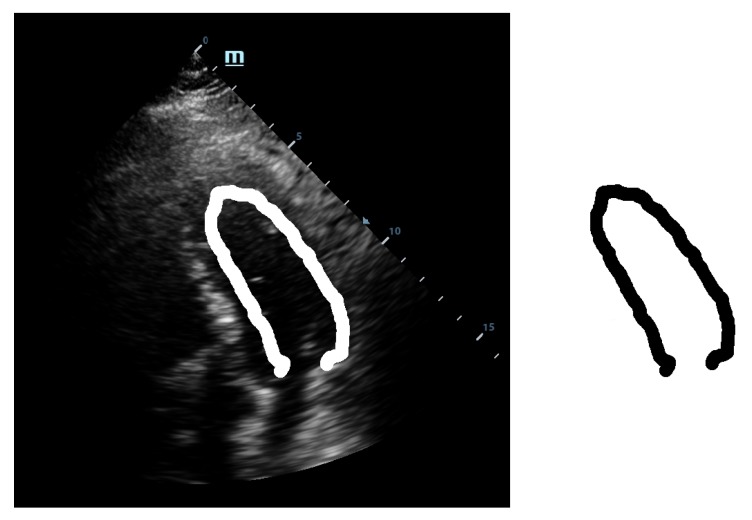
Initial drawing and template extraction.

The first step was a preliminary tracing of the endocardium (very rough drawing) on an ultrasound image done by hand by a trained cardiologist. That was the beginning of our approach. From this initial sketch (drawing), we extracted a draft template (see Figure 1).

**Figure 2:**
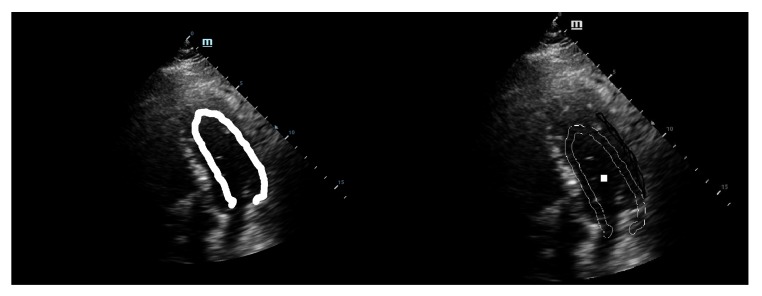
Direct application of the anisotropic Hough transform.

This template was used for the initial tests with our implementation of the GHT. We considered an anisotropic Hough transform to allow deformations of the template that could best fit the left ventricular wall. By directly applying the GHT with this template on an echocardiographic image, we obtained a rough marking of the left ventricular wall. The results are shown in Figure 2.

As shown in Figure 2, the results were similar to the hand drawing by the cardiologist but not satisfactory from the point of view of a precise marking of the left ventricular wall. These results could be used for the training sessions but are not useful in the case of clinical practice. Therefore, we decided to improve and calibrate the detection procedure following two paths: refining the template and detection of the region of interest [15].

**Figure 3:**
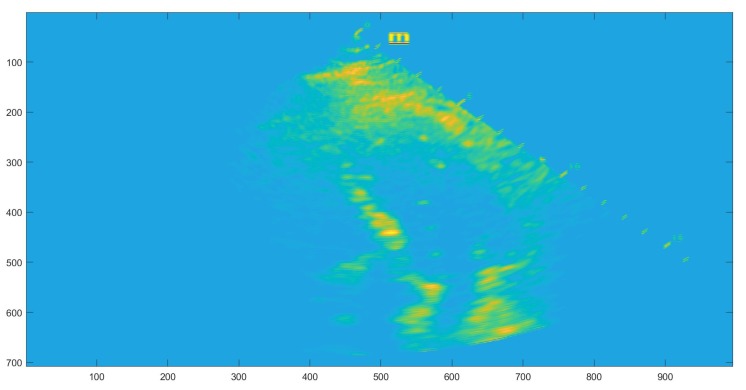
ROI after Gabor-like filter.

The localization of a region of interest (ROI) on the echographic image is of crucial importance for accurate application of the GHT. The correct marking of ROI eliminates most of the detection problems encountered by the GHT. For the detection of the application area, we employed a Gabor-like filtering. The Gabor-like filtering gave us a corresponding region of interest for the application of the GHT (see Figure 3).

**Figure 4:**
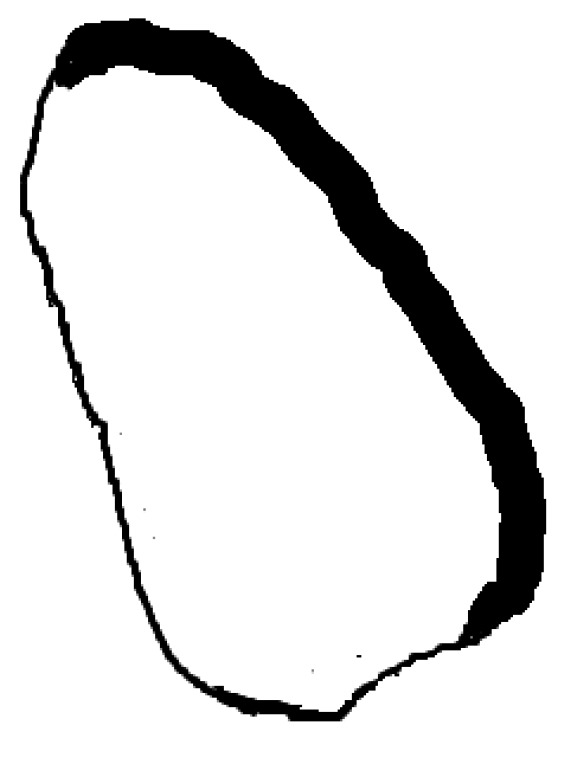
Refined anisotropic template.

Simultaneously, by following the second path, we decided to also make also the template deformable, not only its application to the Hough transform. Therefore, we used the septal wall of the LV as guidance because this side has a higher visibility as it is not usually covered by other anatomical structures such as lungs or bones when observed echographically. To obtain a thinner line, the deformation was made within the original template. This way we managed to obtain the refined deformable template, as shown in Figure 4.

This template was then used as the guidance template. The medial wall was found and initially estimated. Afterwards, by applying the Hough transform, the deformation within the thick guidance template line was performed such that the delimitation of the lateral wall of the LV was obtained as accurately as possible while the rest of the template was thin because its primary use was for orientation purposes inside the echocardiographic image. The guidance template is suitable to adapt and localize within the ROI area of the left ventricle by making use of its visible anatomical features. This is needed also because the algorithm is adaptable to the phases of the heart and a fixed template would have not been able to recognize the boundaries of the LV in a lively-automatic fashion.

**Figure 5:**
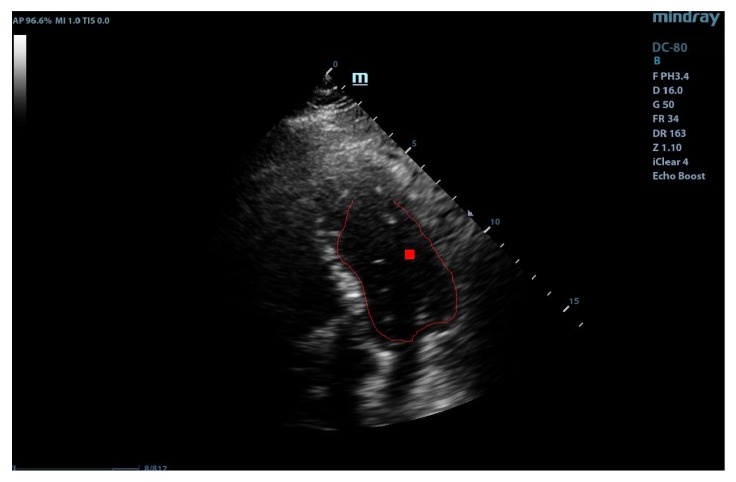
Complete application of the anisotropic Gabor–Hough transform.

By combining the two paths, we obtained the final marking of the left ventricle (see Figure 5). The marking of the left ventricle wall can be observed following the thin red line.

We are now able to provide the full algorithm below in a straightforward implementation format.

The application of the algorithm computes the ten best recognition positions and the selection of the most qualitative marking is done with a rate given by the maxima of the accumulator space.

## 4. Results and Medical Discussions

The algorithm was tested on several sets of ultrasound images provided by the Cardiology Clinic of the County Emergency Hospital Timisoara, the largest medical facility in the western part of Romania. The echographic images were anonymized and no link with the actual patients is possible. For the 18 datasets, we selected a healthy patient cohort without any abnormalities but different physiological and anatomical characteristics such as height, body weight, etc. The dataset is constituted by adult patients, aged between 30–50 years, without cardiovascular diseases in order to avoid the impact of morphological changes (e.g., hypertrophy/dilatation) or functional ones (hypo/a/dyskinesia) on the results of the algorithm. These patients had poor echocardiographic window due to pulmonary pathology or anatomical characteristics.

**Figure 6:**
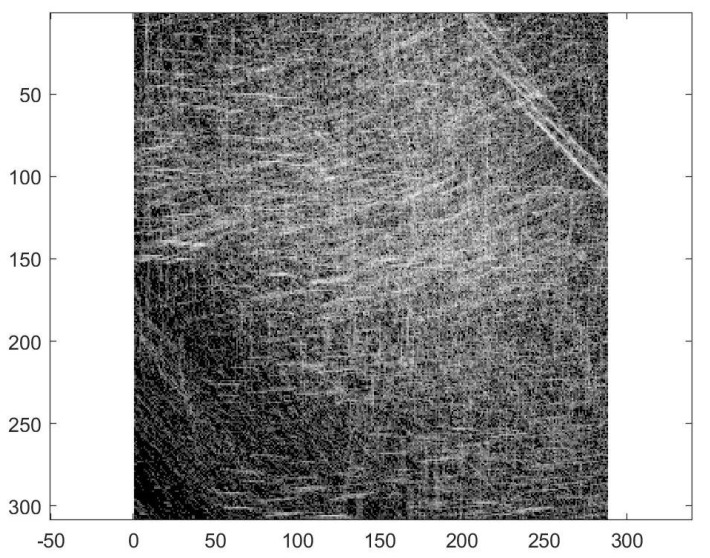
The complete accumulator space.

The application of the complete algorithm already provided us with an estimate of the form: *The best matching was found for scale 0.885000 and angle 1.047 in position (98, 101) with rate 67*. This estimation is directly related with the maxima of the corresponding accumulator space. However, for accuracy reasons, we wanted to provide to the clinical medical personnel an error indicator regarding when the application of the procedure has a prospective positive impact on the precision of the diagnostic. Therefore, we trained a support vector machine classifier (SVM) on the accumulator space. SVM is an established machine learning supervised classifier based on a kernel function and using the well-known kernel trick [16]. We trained the SVM using a cubic kernel function and an automatic kernel scale on the complete accumulator space (see Figure 6). We obtained an accuracy of 64.8% and a training time of 8.35 s using parallel training on a standard notebook with Intel Core i5-6200U Processor, 3 M Cache, and up to 2.80 GHz. This accuracy of classification is by far not competitive compared with other more sophisticated methods from the literature, thus we re-computed the accumulator space after the computation of the Gabor filtering.

**Figure 7:**
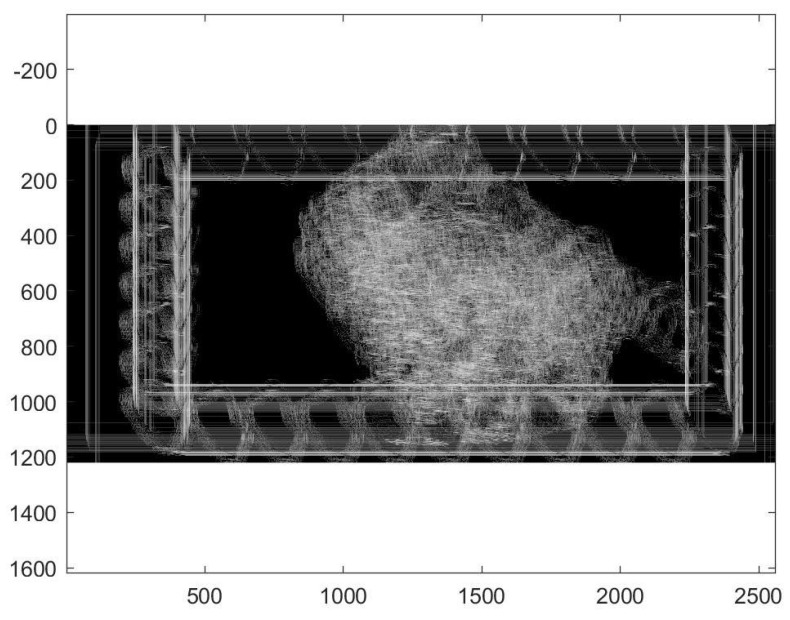
Restricted accumulator space.

The filtered and restricted accumulator space is shown in Figure 7. After applying the anisotropic GHT with the guided deformable template proposed in the previous section, we re-trained the SVM with a cubic kernel function and an automatic kernel scale. We obtained an accuracy of 99.8% and a training time of 19.421 s using parallel training on the same standard notebook with Intel Core i5-6200U Processor, 3M Cache, and up to 2.80 GHz.

Therefore, we conclude that the sequential procedure proposed in Algorithm 1 dramatically improved the application of the Hough transform and no longer needed to compute other anatomical features or volumes compared to similar methods [5,6], making it suitable for prospective clinical investigations in regular medical facilities without high-level hardware equipment and research amenities.
**Algorithm 1:** GHT marking of the left ventricle.
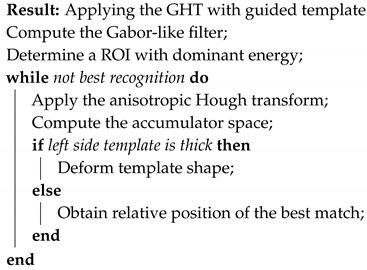


**Figure 8:**
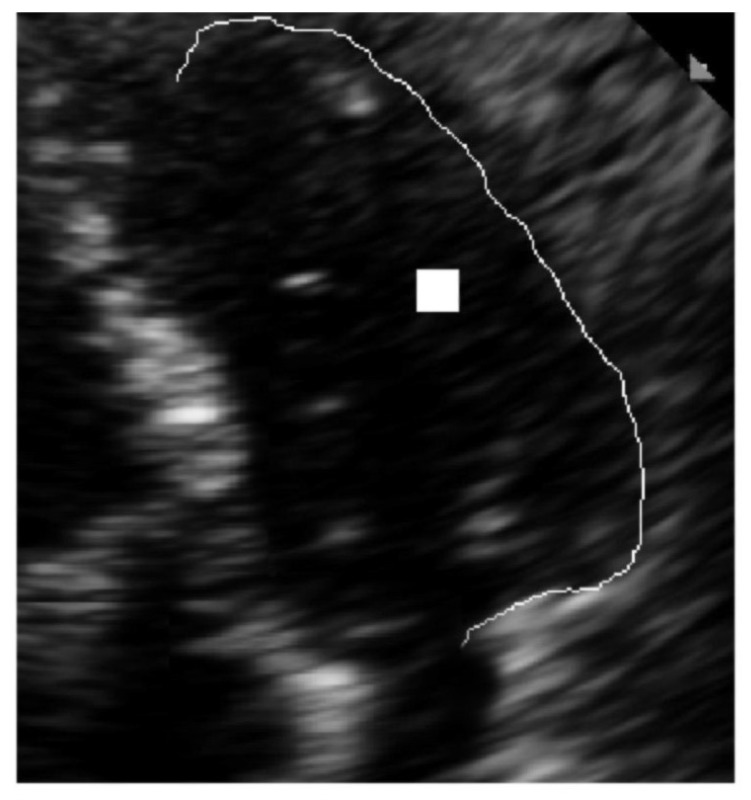
Recognition of the lateral wall of the LV in detail.

The latter recognition is shown in detail in Figure 8. In this figure, only the lateral wall of the LV is marked explicitly because the medial wall of the LV was previously estimated for the template reference system.

The Gabor-filtered GHT algorithm together with the SVM accuracy estimation was tested on a set of 18 ultrasound images. The study case presented is situated in position 7 in the order of accuracy, but some echocardiographic images were of poor quality also for delimiting the medial wall of the LV that was used as guidance.

## 5. Conclusions and Final Discussion

In a series of very recent papers [17,18,19,20], the clinical effects of the left ventricle dysfunction were deeply investigated, making our research algorithm suitable for most of these observed and reported clinical symptoms.

The echocardiographic quantification method recommended by guidelines [2] is the biplane method modified by Simpson, which requires manual tracing of the endocardial border in end-diastole and end-systole in four chamber view. The method is time-consuming, requires visualization of the endocardial border of the entire LV cavity, and requires substantial expertise in obtaining optimal images because, in some cases, atypical views are needed. Obtaining reliable and reproducible information about the dimensions, contractility, and function of cardiac chambers is essential in clinical cardiology, since almost all diagnostic and therapeutic decisions, as well as patient’s prognosis, are based on these data. Echocardiography allows the most convenient and accessible assessment of important hemodynamic parameters, being at the same time inexpensive and non-invasive. It is widely used in clinical practice but there are limitations related to the quality of the acoustic windows, e.g., in overweight patients, in those with obstructive pulmonary disease, or in those on mechanical ventilation where the examination is difficult, EBD is hard to recognize and the best imaging equipment is required, the results being delayed, as well as the appropriate management of the patient. Often, time consuming and expensive diagnostic methods such as MRI and SPECT, or even invasive ones, are required, exposing the patient to an additional risk. In some studies [3,4], it is estimated that about 15% of routine echocardiograms and up to 30% of those performed on patients with poor acoustic window are suboptimal. Reduced EBD represents a pitfall of echocardiogram without contrast.

Compared to other digital image processing techniques that have been developed to overcome these challenges (e.g., [5,6]), we have observed that, to obtain a similar accuracy as our method, they perform a post processing of the echocardiographically acquired images, which is laborious and time consuming. In contrast, the method proposed in this paper has the advantages that it is lively-automatic and therefore it is directly implementable and responsive without the need of computing further anatomical characteristics, e.g., the apex. In addition, through the SVM classifier, it gives an informative estimation on the accuracy and the benefit of its application, which largely varies from patient to patient depending on his anatomy. The cross-validation scheme used for performing the evaluation is the Leave-one-out cross-validation (LOOCV) for a training set of 17 patients from the total of 18. This validation is necessary after the application of the SVM classifier because the validation set and training set are drawn from the same population. We found that the best performance will occur when all 18 features extracted from the test images are used. We are expecting limitations of our algorithm from two sources: the echocardiographic window that sometimes could not provide us with a good reference system for the anisotropic template and the support vector classifier that could overfit the data. To mitigate these limitations of the algorithms, a database of accumulation spaces is currently being built and its processing will be included in the algorithm. In addition, there exists modern ultrasound machines equipped with border recognition software, but their efficiency in patients with pour ecographic windows remains a matter of debate. Our algorithm can improve the images acquired with any echocardiographic machine not only the ones in specialized research facilities.
